# Augmented Pulmonary Responses to Acute Ozone Exposure in Obese Mice: Roles of TNFR2 and IL-13

**DOI:** 10.1289/ehp.1205880

**Published:** 2013-02-22

**Authors:** Alison Suzanne Williams, Joel Andrew Mathews, David Itiro Kasahara, Lucas Chen, Allison Patricia Wurmbrand, Huiqing Si, Stephanie Ann Shore

**Affiliations:** Department of Environmental Health, Harvard School of Public Health, Boston, Massachusetts, USA

**Keywords:** airway responsiveness, bronchoalveolar lavage, IL-5, inflammation, MIP-3α

## Abstract

Background: Acute ozone (O_3_) exposure results in greater inflammation and airway hyperresponsiveness (AHR) in obese versus lean mice.

Objectives: We examined the hypothesis that these augmented responses to O_3_ are the result of greater signaling through tumor necrosis factor receptor 2 (TNFR2) and/or interleukin (IL)-13.

Methods: We exposed lean wild-type (WT) and *TNFR2*-deficient (*TNFR2^–/–^*) mice, and obese *Cpe^fat^* and *TNFR2*-deficient *Cpe^fat^* mice (*Cpe^fat^*/*TNFR2^–/–^*), to O_3_ (2 ppm for 3 hr) either with or without treatment with anti–IL-13 or left them unexposed.

Results: O_3_-induced increases in baseline pulmonary mechanics, airway responsiveness, and cellular inflammation were greater in *Cpe^fat^* than in WT mice. In lean mice, TNFR2 deficiency ablated O_3_-induced AHR without affecting pulmonary inflammation; whereas in obese mice, TNFR2 deficiency augmented O_3_-induced AHR but reduced inflammatory cell recruitment. O_3_ increased pulmonary expression of IL-13 in *Cpe^fat^* but not WT mice. Flow cytometry analysis of lung cells indicated greater IL-13–expressing CD4^+^ cells in *Cpe^fat^* versus WT mice after O_3_ exposure. In *Cpe^fat^* mice, anti–IL-13 treatment attenuated O_3_-induced increases in pulmonary mechanics and inflammatory cell recruitment, but did not affect AHR. These effects of anti–IL-13 treatment were not observed in *Cpe^fat^*/*TNFR2^–/–^* mice. There was no effect of anti–IL-13 treatment in WT mice.

Conclusions: Pulmonary responses to O_3_ are not just greater, but qualitatively different, in obese versus lean mice. In particular, in obese mice, O_3_ induces IL-13 and IL-13 synergizes with TNF via TNFR2 to exacerbate O_3_-induced changes in pulmonary mechanics and inflammatory cell recruitment but not AHR.

Ozone (O_3_), an air pollutant, causes respiratory symptoms and reductions in lung function (Alexis et al. 2000). O_3_ is also a trigger for asthma: Asthma-related emergency room visits increase on days of high ambient O_3_ (Fauroux et al. 2000; Gent et al. 2003). O_3_ activates the innate immune system causing pulmonary infiltration with neutrophils and airway hyperresponsiveness (AHR), a characteristic feature of asthma (Garantziotis et al. 2009).

Two-thirds of the U.S. population is obese or overweight (National Center for Health Statistics 2012), and obesity is a risk factor for asthma (Shore and Johnston 2006). Nevertheless, our understanding of how obesity impacts pulmonary responses to O_3_ is still rudimentary. O_3_-induced decrements in lung function are greater in obese and overweight than lean human subjects (Alexeeff et al. 2007; Bennett et al. 2007). Obese mice also exhibit greater pulmonary inflammation and greater AHR than lean mice after acute O_3_ exposure (Johnston et al. 2006; Shore et al. 2003). The mechanistic basis for the effect of obesity on responses to O_3_ is unknown.

Tumor necrosis factor-α (TNFα) is induced in the lung after O_3_ exposure and has been implicated in responses to acute O_3_ exposure (Cho et al. 2001; Matsubara et al. 2009; Shore et al. 2001; Yang et al. 2005). Serum TNFα increases with obesity (Katsuki et al. 1998; Williams et al. 2012). *TNF*α promoter polymorphisms that augment TNFα expression are associated with an increased obesity-related risk of asthma, especially nonatopic asthma (Castro-Giner et al. 2009), suggesting that TNFα may also be relevant for obesity-related asthma. The role of TNFα in the augmented responses to acute O_3_ observed in obese mice has not been established.

TNFα binds to two receptors, TNFR1 and TNFR2, which differ in their ability to induce inflammation and apoptosis and in their affinity for cleaved versus membrane-associated TNFα (Naude et al. 2011). In lean mice, O_3_-induced AHR requires TNFR2 (Shore et al. 2001), though TNFR1 may also play a role (Cho et al. 2001). TNFR2 is also required for the innate AHR that is observed in obese mice (Williams et al. 2012), but the role of TNFR2 in the augmented responses to O_3_ observed in obese mice is not established.

The first purpose of this study was to examine the hypothesis that TNFR2 is required for the augmented response to acute O_3_ exposure associated with obesity. To examine this hypothesis, we bred *Cpe^fat^* mice that were genetically deficient in the TNFR2 receptor (*Cpe^fat^*/*TNFR2^–/–^* mice). *Cpe^fat^* mice lack carboxypeptidase E (Cpe), an enzyme involved in appetite regulation and energy expenditure (Leibel et al. 1997). Lack of Cpe leads to obesity (Johnston et al. 2006, 2010). We assessed airway responsiveness and pulmonary injury and inflammation in *Cpe^fat^*/*TNFR2^–/–^* mice along with wild-type (WT), *Cpe^fat^*, and *TNFR2^–/–^* mice.

Interleukin (IL)-13 also plays an important role in AHR. In mice, exogenous administration of IL-13 to the lungs results in AHR, and IL-13–blocking reagents inhibit allergen-induced AHR (Grunig et al. 1998; Wills-Karp et al. 1998). IL-13 may also play a role in responses to acute O_3_ exposure: In lean BALB/c mice, IL-13 deficiency reduces O_3_-induced AHR and inflammation (Pichavant et al. 2008; Williams et al. 2008), but the role of IL-13 in responses to O_3_ in obese mice is not established. Consequently, to examine the hypothesis that IL-13 contributes to obesity-related differences in the response to O_3_, we measured IL-13 expression and examined the effects of anti–IL-13 antibodies in obese and lean mice. Because TNF and IL-13 can synergize to promote the expression of chemokines that may contribute to the effects of O_3_ (Reibman et al. 2003), we examined the effect of anti–IL-13 treatment in both *TNFR2*-sufficient and -deficient mice.

## Methods

*Animals.* This study was approved by the Harvard Medical Area Standing Committee on Animals. Animals were treated humanely and with regard for alleviation of suffering. We bred *Cpe^fat^*/TNFR2^–/–^, TNFR2^–/–^, *Cpe^fat^*, and WT mice as previously described (Williams et al. 2012). Female mice were on a C57BL/6 background, were fed standard mouse chow diets, and were 10–12 weeks old.

*Protocol.* We exposed mice to 2 ppm O_3_ for 3 hr. Twenty-four hours after exposure, we measured pulmonary mechanics and airway responsiveness, performed bronchoalveolar lavage (BAL), harvested blood by cardiac puncture, and collected lungs for preparation of RNA. Controls for these mice were not exposed to O_3_ but were otherwise treated identically and studied simultaneously. Pulmonary mechanics and airway responsiveness for these unexposed mice have been previously reported (Williams et al. 2012). We treated other mice with anti–IL-13 antibody (2 μg/g body weight intraperitoneally) 24 hr before O_3_ exposure. In a final cohort of O_3_-exposed mice, we harvested lungs at 24 hr, enzymatically digested the lungs, and isolated lung cells for flow cytometry to quantitate IL-13–expressing CD4^+^ cells.

*Measurement of pulmonary mechanics and airway responsiveness.* We generated quasi static pressure volume (PV) curves, assessed baseline pulmonary mechanics using the forced oscillation technique, and measured airway responsiveness to aerosolized methacholine as previously described (Williams et al. 2012). We assessed Newtonian resistance (Rn), which largely reflects the resistance of the conducting airways, and the coefficients of lung tissue damping (G) and lung tissue elastance (H), which reflect changes in the small airways and pulmonary parenchyma.

*Bronchoalveolar lavage.* We lavaged lungs and counted BAL cells as previously described (Williams et al. 2012). We measured BAL cytokines, chemokines, and hyaluronan by ELISA (R&D Systems Inc., Minneapolis, MN; eBioscience, San Diego, CA; and Echelon Biosciences, Salt Lake City, UT). We measured BAL protein by Bradford assay (BioRad, Hercules, CA).

*RNA extraction and real-time polymerase chain reaction (PCR).* We used real-time PCR to quantitate IL-13 and IL-17A mRNA expression as described (Shore et al. 2011). We subtracted Ct values for a housekeeping gene, *36B4* (*rplp0*) (which codes for a ribosomal protein), from Ct values for IL-13 or IL-17A to obtain ΔCt values. We expressed changes in mRNA relative to values from the WT unexposed mice, using the ΔΔCt method.

*Flow cytometry.* We flushed the lungs to remove blood cells, and then excised, minced, and digested lung tissue as previously described (Kasahara et al. 2012). We cultured lung cells either with or without PMA (phorbol myristate acetate) and ionomycin, in the presence of Golgi Stop (BD Bioscience, Franklin Lakes, NJ), for 5 hr before staining for flow cytometry. Cells were fixed with 4% paraformaldehyde, permeabilized with 0.1% Triton X-100, incubated with anti-Fcγ blocking mAb (clone 93; Biolegend, San Diego, CA), and washed. We stained the cells with Alexa Fluor 488-conjugated CD4 mAb (clone GK1.5; Biolegend) and Alexa Fluor 647-conjugated anti-mouse IL-13 (clone eBio13A; eBioscience). We passed the cells through a BD Canto flow cytometer (BD Bioscience), and analyzed the data with FlowJo software (Tree Star Inc., Ashland, OR).

*Statistics.* We used factorial analysis of variance to assess the significance of differences in outcome indicators, as previously described (Williams et al. 2012). We performed analyses using Statistica version 6 (StatSoft, Tulsa, OK).

## Results

*Body mass.* There was a significant effect of *Cpe* (*p* < 0.001) but not *TNFR2* genotype on body mass. Whether or not they were deficient in TNFR2, *Cpe^fat^* mice weighed about twice as much as lean controls, consistent with previous observations (Williams et al. 2012).

**Figure 1:**
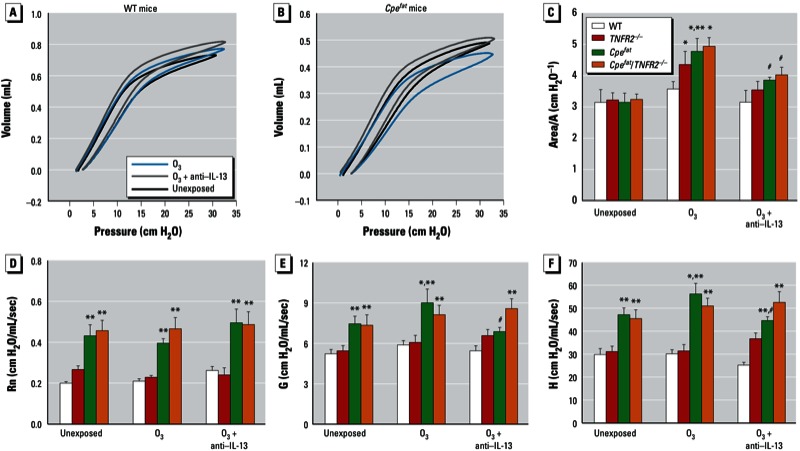
O_3_-induced changes in pulmonary mechanics of mice 24 hr after O_3_ exposure. Mice were unexposed, exposed to O_3_, or treated with anti–IL-13 24 hr before O_3_ exposure. Pressure volume (PV) curves of WT (*A*) and *Cpe^fat^* (*B*) mice. The areas of the PV curves were normalized for volume A (the differences between total lung capacity and end expiratory volume, i.e., the height of the PV curves) (*C*), baseline values for airway resistance (Rn) (*D*), the coefficient of lung tissue damping, G (*E*), and the coefficient of lung tissue elastance, H (*F*). Values shown are mean ± SE of data from 6–9 mice in each group. *p < 0.05, compared with unexposed genotype-matched mice. **p < 0.05, compared with TNFR2 genotype-matched lean mice with the same exposure. #*p* < 0.05, compared with O_3_-exposed genotype-matched mice not treated with anti–IL-13.

*Pulmonary mechanics and airway responsiveness.* In WT mice, no significant differences in the PV curve of the lungs were observed in O_3_-exposed versus unexposed mice (Figure 1A). However, in *Cpe^fat^* mice, there was a rightward shift and widening of the PV loop indicative of increased hysteresis in O_3_-exposed versus unexposed mice (Figure 1B). To quantitate these changes, we measured the area of the PV loop, and normalized it by A, the difference in volume between total lung capacity and end expiratory lung volume (height of the PV loops in Figure 1). Area/A is a measure of the thickness of the PV loop. A was lower in obese versus lean mice, as previously reported (Williams et al. 2012), but there was no difference in A in O_3_-exposed versus unexposed mice (data not shown). Consistent with the increased hysteresis induced by O_3_ in *Cpe^fat^* but not WT mice (Figure 1A,B), Area/A was greater in O_3_-exposed versus unexposed *Cpe^fat^* mice, whereas O_3_ had no effect on Area/A in WT mice (Figure 1C). Area/A was also greater in O_3_-exposed versus unexposed *Cpe^fat^*/*TNFR2^–/–^* mice (Figure 1C).

Even in unexposed mice, baseline Rn, G, and H were elevated in the lungs of obese mice (Figure 1D,E,F). O_3_ exposure had no effect on Rn in mice of any genotype, but O_3_ exposure increased baseline G and H in *Cpe^fat^* mice (Figure 1D,E,F). In contrast, no significant changes in baseline G and H were observed in O_3_-exposed versus unexposed *Cpe^fat^*/*TNFR2^–/–^* mice.

**Figure 2:**
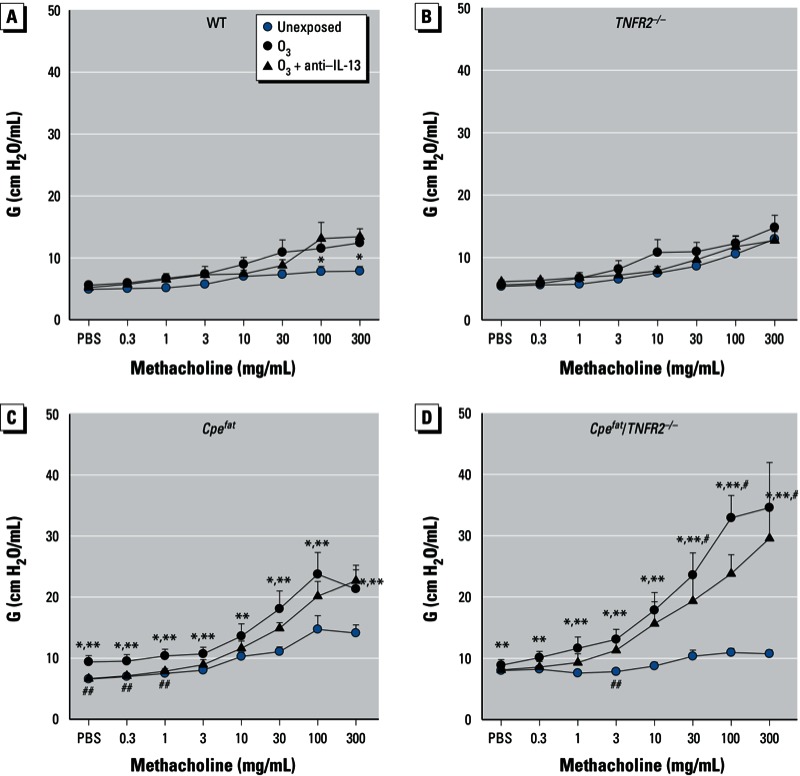
Airway responsiveness of mice 24 hr after exposure. Mice were unexposed, exposed to O_3_, or treated with anti–IL-13 24 hr before O_3_ exposure. Methacholine-induced changes in G, a measure of the lung tissue response in WT (*A*), TNFR2^–/–^ (*B*), *Cpe^fat^* (*C*), and *Cpe^fat^*/TNFR2^–/–^ (*D*) mice. Values shown are mean + SE of data from 6–9 mice per group. *p < 0.05, O_3_-exposed compared with unexposed genotype-matched mice. **p < 0.05, compared with TNFR2 genotype-matched mice with the same exposure. ^#^*p* < 0.05, compared with obesity-matched TNFR2-sufficient mice with the same O_3_ exposure. ^##^*p* < 0.05, compared with O_3_-exposed genotype-matched mice not treated with anti–IL-13.

Airway responsiveness was greater in unexposed *Cpe^fat^* versus WT mice, and this difference was abolished by TNFR2 deficiency (see Williams et al. 2012). In lean WT mice, O_3_ exposure caused AHR (Figure 2A). This effect of O_3_ was observed when G, a measure of the lung tissue response, was used as the outcome indicator (Figure 2A). A similar trend was observed for H, but did not reach statistical significance [see Supplemental Material, Figure S1 (http://dx.doi.org/10.1289/ehp.1205880)]. There was no effect of O_3_ on methacholine-induced changes in Rn (see Supplemental Material, Figure S1A). O_3_-induced AHR was absent in *TNFR2^–/–^* mice (Figure 2B). O_3_ did cause AHR in *Cpe^fat^* mice (Figure 2C), and the magnitude of the O_3_-induced AHR was significantly greater than in WT mice. O_3_ also induced AHR in *Cpe^fat^*/*TNFR2^–/–^* mice (Figure 2D). Indeed, O_3_-induced AHR was actually greater in *Cpe^fat^*/TNFR2^–/–^ than in *Cpe^fat^* mice, and O_3_-induced AHR was observed even when changes in Rn were used as the outcome indicator, whereas this was not the case in mice of any other genotype (see Supplemental Material, Figure S1D). O_3_-induced AHR was also observed in *Cpe^fat^*/TNFR2^–/–^ and *Cpe^fat^* mice when H was used as the index of response, and AHR based on changes in H was also greater in *Cpe^fat^*/TNFR2^–/–^ than in *Cpe^fat^* mice (see Supplemental Material, Figure S1G,H).

**Figure 3:**
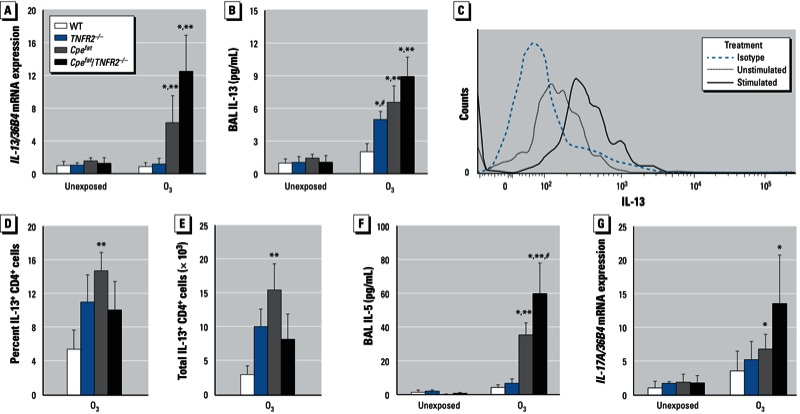
*IL-13* mRNA expression in lungs (*A*) and IL-13 concentration in BAL (*B*) from mice that were either unexposed or exposed to O_3_. Example of flow cytometry data from one *Cpe^fat^* mouse (*C*) showing histograms of CD4^+^ lung cells stained with isotype control antibody or stained with anti–IL-13 and either unstimulated or stimulated with PMA (10 ng/mL) and ionomycin (500 ng/mL) for 5 hr to induce cytokine expression. Shown also are the percentages (*D*) and total number (*E*) of PMA and ionomycin stimulated CD4^+^ cells isolated from lungs of O_3_-exposed mice that expressed IL-13. BAL IL-5 concentrations (*F*) and *IL-17A* mRNA expression (*G*) in unexposed and O_3_-exposed mice. Values shown are mean ± SE of data from 4–8 mice per group. Results for *IL-13* and *IL-17A* mRNA are normalized to 36B4 expression. *p < 0.05, compared with unexposed genotype-matched mice. **p < 0.05, compared with *TNFR2* genotype-matched lean mice with the same exposure. ^#^*p* < 0.05, compared with obesity-matched *TNFR2*-sufficient mice with the same O_3_ exposure.

Because IL-13 has been shown to contribute to O_3_-induced AHR in lean BALB/c mice (Pichavant et al. 2008; Williams et al. 2008), we determined whether there were obesity- and/or TNFR2-dependent differences in IL-13 expression. O_3_ caused an increase in *IL-13* mRNA in obese but not lean mice (Figure 3A). There was a nonsignificant trend toward increased *IL-13* mRNA expression in *Cpe^fat^*/TNFR2^–/–^ versus *Cpe^fat^* mice. O_3_ exposure also increased BAL IL-13 in *Cpe^fat^* but not WT mice (Figure 3B). Furthermore, both the percentage and the total number of IL-13–expressing CD4^+^ cells were higher in *Cpe^fat^* than WT mice exposed to O_3_ (Figure 3D,E). Consequently, we also measured BAL concentrations of another Th2 cytokine, IL-5. O_3_-induced increases in IL-5 were significantly greater in obese versus lean mice, and TNFR2 deficiency significantly increased BAL IL-5 levels in obese but not lean mice (Figure 3F), consistent with the trend observed for IL-13 (Figure 3B). IL-17A has also been linked to AHR (Kudo et al. 2012). O_3_ exposure caused a significant increase in *IL-17A* mRNA in obese but not lean mice (Figure 3G). There was a nonsignificant trend toward greater IL-17A in *Cpe^fat^*/TNFR2^–/–^ than *Cpe^fat^* mice.

To examine the functional impact of this IL-13 expression, we treated mice with antibodies to IL-13. In WT mice, there was no effect of anti–IL-13 treatment, likely because pulmonary IL-13 was negligible in these mice (Figure 3A,B). In *Cpe^fat^* mice, anti–IL-13 treatment reversed the effects of O_3_ exposure on the PV curve (Figure 1B,C), and it prevented the O_3_-induced increase in baseline G and H (Figure 1 E,F) but did not affect O_3_-induced AHR (Figure 2C). In *Cpe^fat^/TNFR2^–/–^* mice, anti–IL-13 treatment also prevented O_3_-induced changes in the PV curve (Figure 1C) but had no effect on baseline pulmonary mechanics or O_3_-induced AHR (Figure 2D).

**Figure 4:**
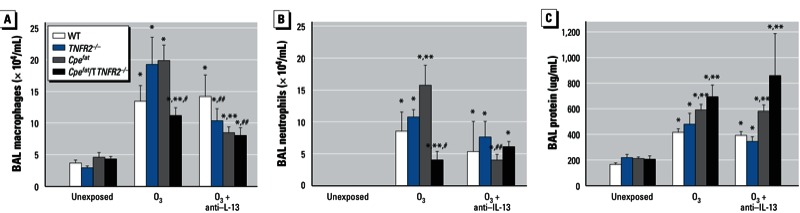
Macrophages (*A*), neutrophils (*B*), and protein (*C*) in BAL from mice that were unexposed, exposed to O_3_, or treated with anti–IL-13 24 hr before O_3_ exposure. Values shown are mean ± SE of data from 4–9 mice per group. *p < 0.05, compared with unexposed genotype-matched mice. **p < 0.05, compared with *TNFR2* genotype-matched lean mice with the same exposure. ^#^*p* < 0.05, compared with obesity-matched *TNFR2*-sufficient mice with the same O_3_ exposure. ^##^*p* < 0.05, compared with O_3_-exposed genotype-matched mice not treated with anti–IL-13.

**Figure 5:**
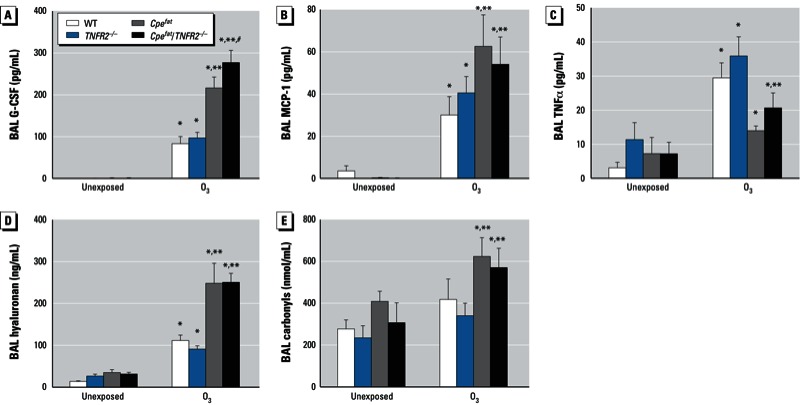
BAL G-CSF (*A*), MCP-1 (*B*), TNFα (*C*) hyaluronan (*D*), protein carbonyls (*E*) from mice that were either unexposed or exposed to O_3_. Values shown are mean ± SE of data from 4–10 mice per group. *p < 0.05, compared with unexposed genotype-matched mice. **p < 0.05, compared with *TNFR2* genotype-matched lean mice with the same exposure. ^#^*p* < 0.05, compared with obesity-matched *TNFR2*-sufficient mice with the same O_3_ exposure.

*Pulmonary inflammation and injury.* O_3_ exposure significantly increased BAL macrophages, neutrophils, and protein [a marker of O_3_-induced injury (Bhalla 1999)] (Figure 4). Lymphocytes and eosinophils were not observed in BAL fluid of most mice regardless of exposure, genotype, or obesity status. BAL neutrophils and protein were increased in *Cpe^fat^* versus WT mice exposed to O_3_ (Figure 4). In lean mice, TNFR2 deficiency had no effect on these responses to O_3_. However, in obese mice, BAL neutrophils and macrophages, but not BAL protein, were significantly reduced in *TNFR2*-deficient versus -sufficient mice (Figure 4). The TNFR2-dependent changes in neutrophil recruitment were not the result of differences in the expression of IL-6, KC (keratinocyte chemoattractant), or G-CSF (granulocyte colony stimulating factor) [Figure 5A; see also Supplemental Material, Figure S2 (http://dx.doi.org/10.1289/ehp.1205880)], cytokines and chemokines reported to be important for O_3_-induced neutrophil recruitment to the lungs (Johnston et al. 2005a, 2005b; Kasahara et al. 2012). These chemokines were greater in O_3_-exposed obese versus lean mice, but they were either not affected (i.e., IL-6 and KC) or actually augmented (i.e., G-CSF) by TNFR2 deficiency in obese mice. Similarly, TNFR2-dependent changes in macrophage recruitment are not the result of changes in MCP-1, a chemokine required for O_3_-induced macrophage recruitment (Zhao et al. 1998). BAL MCP-1 was greater in obese versus lean mice, but TNFR2 deficiency had no effect on BAL MCP-1 (Figure 5B). Surprisingly, O_3_-induced increases in BAL TNFα were significantly reduced in obese versus lean mice (Figure 5C).

O_3_ induces fragmentation of the matrix glycoprotein, hyaluronan, leading to AHR (Garantziotis et al. 2009). Such hyaluronan fragments are thought to be induced via oxidative stress caused by O_3_. O_3_ exposure caused an increase in BAL hyaluronan (Figure 5D) as well as protein carbonyls (Figure 5E), a marker of oxidative stress, although protein carbonyls were only increased in obese and not lean mice. Furthermore, O_3_-induced increases in BAL hyaluronan and protein carbonyls were significantly greater in obese than lean mice (Figure 5D,E), suggesting that elevations in BAL hyaluronan may contribute to greater O_3_-induced AHR in obese versus lean mice. However, BAL hyaluronan does not appear to account for the greater O_3_-induced AHR in *Cpe^fat^*/TNFR2^–/–^ versus *Cpe^fat^* because BAL hyaluronan was not different in these two strains (Figure 5D).

**Figure 6:**
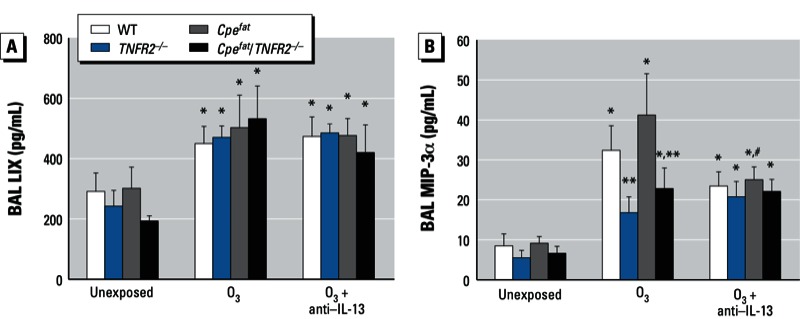
BAL LIX (*A*) and MIP-3α (*B*) from mice that were unexposed, exposed to O_3_, or treated with anti–IL-13 24 hr before O_3_ exposure. Values shown are mean ± SE of data from 5–8 mice per group. *p < 0.05, compared with unexposed genotype-matched mice. **p < 0.05, compared with obesity-matched *TNFR2*-sufficient mice with the same O_3_ exposure. ^#^*p* < 0.05, compared with O_3_-exposed genotype-matched mice not treated with anti–IL-13.

In *Cpe^fat^* mice, anti–IL-13 treatment reduced O_3_-induced increases in BAL neutrophils and macrophages (Figure 4A,B), but not BAL protein (Figure 4C), whereas there was no effect of anti–IL-13 treatment in *Cpe^fat^/TNFR2^–/–^* mice (Figure 4A,B). O_3_-induced increases in *MIP-3*α (*CCL20*; macrophage inflammatory protein 3 α) and *LIX* (*CXCL5*; lipopolysaccharide-induced CXC chemokine) mRNA are reduced in IL-13–deficient versus WT BALB/c mice, suggesting that changes in these chemokines may contribute to the effects of IL-13 deficiency on inflammatory cell recruitment (Williams et al. 2008). O_3_-induced increases in BAL LIX, a neutrophil chemotactic factor, were not affected by obesity, TNFR2 deficiency, or anti–IL-13 treatment (Figure 6A) indicating that this chemokine does not account for the observed effects on BAL neutrophils (Figure 4B). However, TNFR2 deficiency did attenuate O_3_-induced increases in MIP-3α regardless of obesity status (*p* < 0.001) (Figure 6B). Anti–IL-13 also caused a significant reduction in MIP-3α in *TNFR2*-sufficient (*p* < 0.01) but not -deficient mice (Figure 6B), suggesting that IL-13 and TNFα synergize in the induction of MIP-3α after O_3_ exposure.

## Discussion

We observed greater effects of acute O_3_ exposure in obese versus lean mice consistent with previous observations (Johnston et al. 2006; Shore et al. 2003). Importantly, our results also indicated obesity-related differences in the mechanisms governing the pulmonary effects of O_3_ (see Table 1 for summary). In lean mice, TNFR2 deficiency reduced O_3_-induced AHR but had no effect on O_3_-induced inflammation; whereas in obese mice, TNFR2 deficiency enhanced O_3_-induced AHR while attenuating O_3_-induced inflammation (Figures 2,4). In lean mice, IL-13 was not induced by O_3_, and there was no impact of anti–IL-13 treatment on responses to O_3_. However, in obese mice, O_3_ did induce IL-13 expression (Figure 3). Importantly, obesity-related differences in IL-13 expression accounted for the ability of O_3_ to induce changes in the PV curve of lung and to increase baseline G and H in obese *Cpe^fat^* but not lean WT mice (Figure 1). IL-13 also contributed to the greater recruitment of inflammatory cells to the lungs of *Cpe^fat^* versus WT mice (Figure 4). Moreover, in obese mice, IL-13 and TNFR2 appeared to synergize to exacerbate O_3_-induced inflammation and changes in pulmonary mechanics because anti–IL-13 treatment reduced inflammatory cell recruitment and G and H in *TNFR2*-sufficient but not -deficient *Cpe^fat^* mice (Figure 4). MIP-3α expression may have contributed to the TNFR2/IL-13 synergy that promoted inflammatory cell recruitment because TNFR2 deficiency attenuated O_3_-induced increases in BAL MIP3α in untreated mice but not in mice treated with anti–IL-13 (Figure 6). Thus, obesity-related differences in the induction of IL-13 after O_3_ exposure not only confer unique and/or augmented responses to O_3_ on the obese mice, but they also appear to account for some of the obesity-related differences in the impact of TNFR2 deficiency in obese mice because TNFR2 can synergize with IL-13 in obese but not lean mice.

**Table 1:** Effect of IL-13 blockade or TNFR2 deficiency on O_3_-induced increases in airway responsiveness, BAL neutrophil numbers, and pulmonary mechanics in lean and obese mice.

Intervention	Lean mice			Obese mice		
	Airway responsiveness	BAL neutrophils	Pulmonary mechanics	Airway responsiveness	BAL neutrophils	Pulmonary mechanics
IL-13 blockade	No change	No change	No change	No change	Decrease	Decrease
TNFR2 deficiency	Decrease	No change	No change	Increase	Decrease	Decrease
Outcomes (no change, increase, decrease) indicate the qualitative change in the response to O3 relative to the absence of IL-13 blockade or TNFR2 deficiency.						

Our results demonstrating O_3_-induced AHR in WT but not *TNFR2^–/–^* mice (Figure 2) confirm previous reports indicating that TNFR2 is required for O_3_-induced AHR in lean mice (Cho et al. 2001; Shore et al. 2001). Circulating TNFα is increased in obese versus lean mice and the innate AHR characteristic of obese mice is reduced when these mice are TNFR2 deficient (Williams et al. 2012) (the unexposed mice in Figure 2C,D). Hence, we expected that TNFR2 deficiency might also attenuate O_3_-induced AHR in obese mice and might even ablate obesity-related differences in the impact of O_3_ on AHR. We did observe a reduction in O_3_-induced changes in baseline pulmonary mechanics in *Cpe^fat^*/TNFR2^–/–^ versus *Cpe^fat^* mice (Figure 1E,F). However, O_3_-induced AHR was actually greater in *Cpe^fat^*/TNFR2^–/–^ versus *Cpe^fat^* mice (Figure 2). This greater O_3_-induced AHR was not the result of greater obesity in the *Cpe^fat^*/*TNFR2^–/–^* mice: Body mass was the same in the two groups. O_3_-induced increases in BAL TNFα were lower in *Cpe^fat^* versus WT mice (Figure 5C). While this reduction might explain a reduced impact of TNFR2 deficiency in obese mice, it cannot explain the observed reversal in the direction of the impact of TNFR2 deficiency on O_3_-induced AHR.

Because others have reported reduced O_3_-induced AHR in lean IL-13^–/–^ versus WT BALB/c mice and greater O_3_-induced AHR in IL-13 transgenic mice (Pichavant et al. 2008; Williams et al. 2008), we examined the role of IL-13 in O_3_-induced AHR in obese mice. O_3_ caused a significant increase in pulmonary IL-13 mRNA expression and BAL IL-13 in *Cpe^fat^* but not WT mice (Figure 3A,B). CD4^+^ T cells appeared to be the source of this IL-13 (Figure 3C-E), and another CD4^+^ T-cell–derived cytokine, IL-5, was also induced by O_3_ exposure in obese mice (Figure 3F).

To determine whether IL-13 contributed to obesity- and/or TNFR2-dependent changes in the response to O_3_, we treated mice with anti–IL-13 before O_3_ exposure. Anti–IL-13 attenuated the O_3_-induced increase in the hysteresis of the PV curve that was induced by O_3_ exposure in obese mice (Figure 1B,C). Lung surfactant is important in limiting lung hysteresis and IL-13 reduces the pulmonary expression of surfactant protein C (Ito and Mason 2010), which is important for the surface activity of surfactant. Coupled with the observations that O_3_-induced changes in lung hysteresis were not observed in WT mice (Figure 1A,C), which lacked IL-13 (Figure 3), the results suggest that O_3_ caused changes in pulmonary surfactant activity in *Cpe^fat^* mice and that these changes were mediated by IL-13. Changes in lung hysteresis in obese mice may be aggravated by the ability of O_3_ exposure to cause oxidation of phospholipids important for surfactant activity (Pulfer and Murphy 2004). O_3_-induced changes in baseline G and H, measures of the lung tissue, were also ablated by anti–IL-13 treatment (Figure 1). Thus obesity-related differences in the ability of O_3_ to induce IL-13 expression (Figure 3) appear to account for the ability of O_3_ to induce changes in baseline pulmonary mechanics in *Cpe^fat^* but not WT mice. TNFR2 deficiency also reduced O_3_-induced changes in pulmonary mechanics in *Cpe^fat^* mice, and anti–IL-13 treatment had no effect on mechanics in *Cpe^fat^*/*TNFR2^–/–^* mice (Figure 1), suggesting that synergy between IL-13 and TNFR2 contributes to these changes.

In contrast to the effects on baseline pulmonary mechanics (Figure 1), we observed no effect of anti–IL-13 treatment on O_3_-induced AHR in *Cpe^fat^* mice (Figure 2C), nor was there any effect on AHR in *Cpe^fat^*/*TNFR2^–/–^* mice (Figure 2D), indicating that the enhanced AHR in *Cpe^fat^*/*TNFR2^–/–^* mice was not the result of increased IL-13 signaling. Thus, other factors must account for obesity-related increases in O_3_-induced AHR and for the augmented AHR observed in *Cpe^fat^*/*TNFR2^–/–^* mice. IL-5 can induce AHR even in the absence of eosinophils (Borchers et al. 2001) (eosinophils were not observed in these animals). IL-5 was augmented in O_3_-exposed *Cpe^fat^* versus WT mice and further augmented in *Cpe^fat^*/*TNFR2^–/–^* mice (Figure 3F), consistent with the changes in AHR (Figure 2). IL-17A can also induce AHR (Kudo et al. 2012). IL-17A was expressed after O_3_ exposure (Figure 3G), especially in the obese mice, and could also contribute to the augmented O_3_-induced AHR observed in these mice (Figure 2).

In lean mice, TNFR2 deficiency had no effect on O_3_-induced inflammatory cell recruitment (Figure 4), consistent with previous observations (Cho et al. 2001; Shore et al. 2001). In contrast, in obese mice, TNFR2 deficiency reduced O_3_-induced neutrophil and macrophage recruitment and ablated obesity-related differences in these outcomes (Figure 4). Anti–IL-13 treatment also had no effect on O_3_-induced inflammation in lean WT mice, which lacked IL-13 (Figure 3A,B), but anti–IL-13 treatment significantly reduced O_3_-induced inflammatory cell recruitment in obese *Cpe^fat^* mice (Figure 4A,B). A similar reduction in O_3_-induced inflammation occurs in lean IL-13–deficient BALB/c mice (Williams et al. 2008), which are more Th2 prone than the C57BL/6 mice used in the present study. Importantly, in obese mice, TNFR2 and IL-13 appeared to interact to promote inflammatory cell recruitment because anti–IL-13 treatment had no effect on BAL neutrophils or macrophages in *Cpe^fat^*/*TNFR2^–/–^* mice even though these mice had at least as much IL-13 expression as *Cpe^fat^* mice (Figure 4A,B). This interaction may occur at the level of MIP-3α expression. In obese *Cpe^fat^* mice, both TNFR2 deficiency and anti–IL-13 treatment resulted in a significant decrease in BAL MIP-3α (Figure 6B), consistent with observations of others that TNFα and IL-13 can both induce the expression of MIP-3α in airway epithelial cells (Reibman et al. 2003). Importantly, anti–IL-13 treatment inhibited MIP-3α expression in *TNFR2*-sufficient but not -deficient *Cpe^fat^* mice (Figure 6B). Dendritic cells and T cells typically express CCR6, the receptor for MIP-3α, but neutrophils can be induced to express CCR6 in the presence of TNFα (Yamashiro et al. 2000). In addition, MIP-3α can induce migration of IL-17A expressing cells that may contribute to neutrophil recruitment (Li et al. 2011). IL-17A was induced by O_3_ (Figure 3G).

O_3_-induced changes in lung function (i.e., G and H) and cellular inflammation occurred in concert (Table 1). O_3_-induced increases in BAL neutrophils and in both G and H were greater in *Cpe^fat^* versus WT mice. Furthermore, both TNFR2 deficiency and anti–IL-13 treatment reduced O_3_-induced changes in G and H as well as BAL neutrophil numbers in obese mice (Table 1). The results suggest that effects of O_3_ on lung function and inflammation may be mechanistically related. In contrast, O_3_-induced AHR and inflammation were dissociated. For example, TNFR2 deficiency and anti–IL-13 treatment either augmented or had no effect on O_3_-induced mice, but they attenuated O_3_-induced inflammation in obese mice.

Two technical issues require consideration. First, obese mice have a slightly higher minute ventilation (Shore and Johnston 2006) and consequently a slightly higher inhaled dose of O_3_. Their lungs are also smaller (Williams et al. 2012), so the dose per gram of lung tissue may be higher. We do not think this issue contributed substantially to the outcome because neither TNFR2 deficiency nor anti–IL-13 treatment had any effect on lung volume (data not shown), but both reduced the augmented effects of O_3_ on BAL cells and pulmonary mechanics in obese mice. Second, we used the same volume of fluid for lavage in obese and lean mice. Because the lungs of the obese mice were smaller and presumably had correspondingly less lung lining fluid, there should have been greater dilution of substances in that lining fluid and thus lower concentrations of BAL moieties in the obese than the lean mice. In fact, the opposite was true for most BAL cytokines/chemokines examined [Figures 3,5,6; see also Supplemental Material, Figure S2 (http://dx.doi.org/10.1289/ehp.1205880)]. Thus, issues related to normalization of the BAL procedure are unlikely to explain the observed results.

## Conclusions

Our results indicate differences in the mechanisms regulating pulmonary responses to O_3_ in lean and obese mice. In particular, TNFR2 deficiency had opposing effects on O_3_-induced AHR in lean and obese mice. In addition, O_3_ increased the pulmonary expression of IL-13 in obese but not lean mice, and this IL-13 appeared to account for the augmented ability of O_3_ to induce changes in pulmonary mechanics and inflammation in obese mice via synergistic effects with TNFR2. The majority of the population of the United States is either obese or overweight. Our results emphasize the need for improved understanding of the effects of O_3_ in this population.

## Supplemental Material

(520 KB) PDF
