# Supplementary material for: Augmented Pulmonary Responses to Acute Ozone Exposure in Obese Mice: Roles of TNFR2 and IL-13
Source: Environ Health Perspect. 2013 Feb 22;121(5):551–7. doi: 10.1289/ehp.1205880 (PMC3673194; doi:10.1289/ehp.1205880)
Supplement: (520 KB) PDF [file ehp.1205880.s001.pdf]

Supplemental Material

Augmented Pulmonary Responses to Acute Ozone Exposure in Obese Mice: Roles of TNFR2 and IL-13

Alison Suzanne Williams, Joel Andrew Mathews, David Itiro Kasahara, Lucas Chen, Allison Patricia Wurmbrand, Huiqing Si, and Stephanie Ann Shore

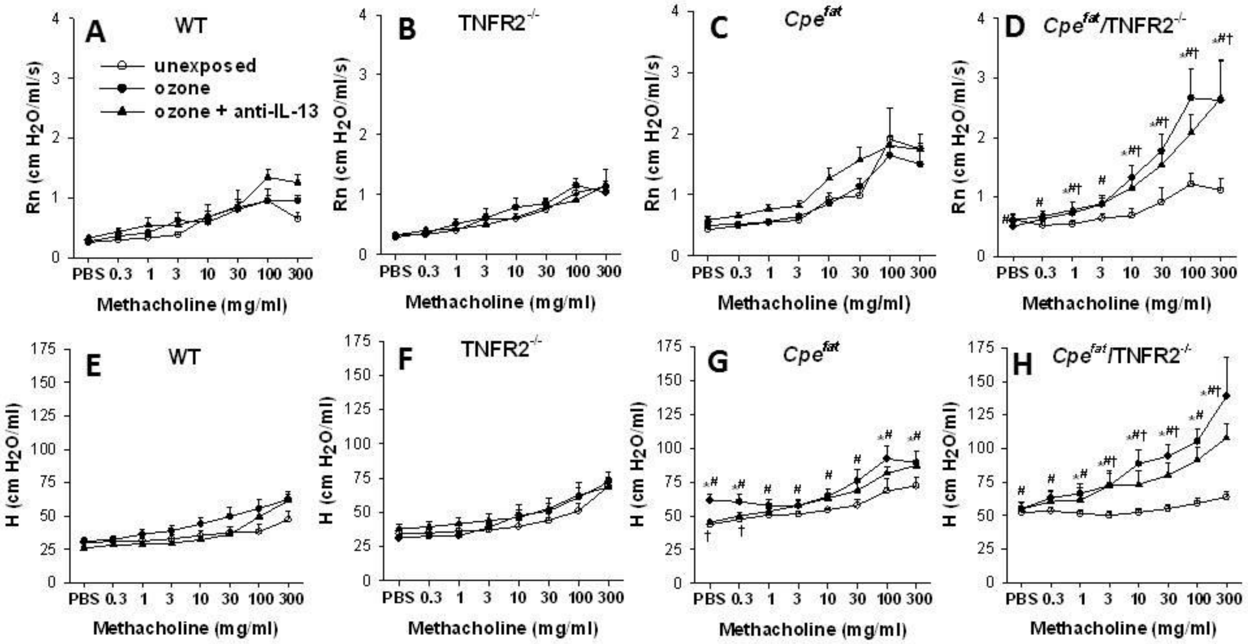

Supplemental Material, Figure S1 Airway responsiveness in mice that were unexposed, exposed to ozone, or treated with anti-IL-13 24 h prior to ozone exposure. Shown are methacholine induced changes in Rn (A-D), which largely reflects the conducting airways, and the coefficient of lung tissue elastance (H) (E-H), which reflects changes in the small airways and pulmonary parenchyma, including airway closure. Results are mean $\pm$ SE of data from 6-9 mice per group. * $p<0.05$ for ozone exposed versus unexposed genotype-matched lean mice. #$p<0.05$ versus TNFR2 genotype matched lean mice with the same exposure; † $p<0.05$ versus obesity-matched TNFR2 sufficient mice with the same ozone exposure; & $p<0.05$ versus ozone exposed genotype matched mice not treated with anti-IL-13.

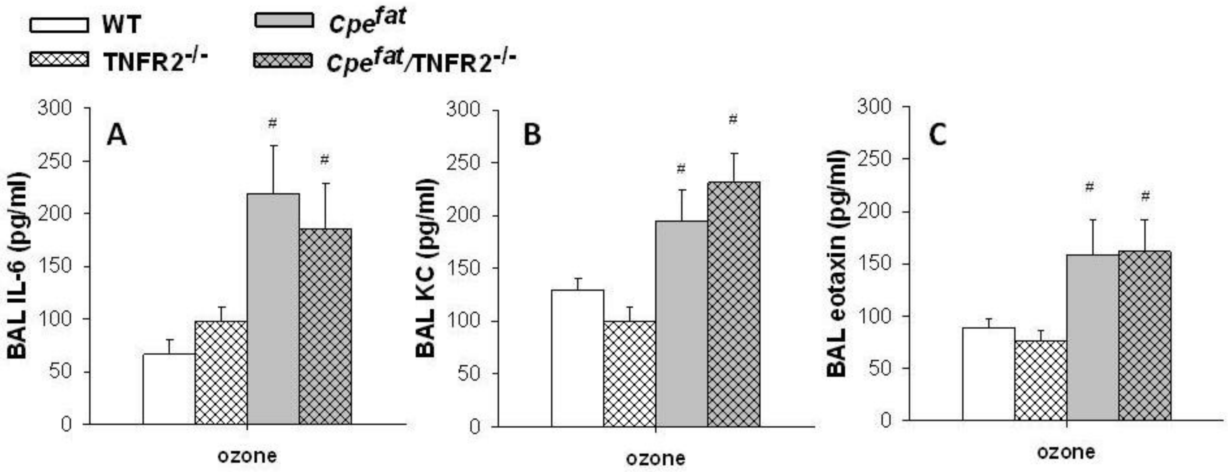

Supplemental Material, Figure S2: Bronchoalveolar lavage (BAL) IL-6 (A), KC (B), and eotaxin (C) in WT, TNFR2$^{-/-}$, *Cpe*^{fat}, and *Cpe*^{fat}/TNFR2$^{-/-}$ mice that were exposed to ozone (2 ppm for 3 h) and studied 4 h after cessation of exposure. We measured IL-6, KC, and eotaxin 4h rather than 24 h after cessation of ozone exposure, because these cytokines/chemokines peak at 4h and then decline towards unexposed values by 24 h. Results are mean $\pm$ SE of data from 5-11 mice per group. #$p<0.05$ versus ozone exposed and TNFR2 genotype matched lean mice.
